# Chirurgie periampullärer Pankreaskarzinome

**DOI:** 10.1007/s00104-021-01462-1

**Published:** 2021-07-14

**Authors:** Thomas Hank, Ulla Klaiber, Klaus Sahora, Martin Schindl, Oliver Strobel

**Affiliations:** Klinik für Allgemeinchirurgie, Abteilung für Viszeralchirurgie, Medizinische Universität Wien, Währinger Gürtel 18–20, 1090 Wien, Österreich

**Keywords:** Periampulläre Tumoren, Duktales Adenokarzinom des Pankreas, Duodenopankreatektomie, Adjuvante Therapie, Ampulläre Karzinome, Periampullary tumors, Pancreatic ductal adenocarcinoma, Pancreatoduodenectomy, Adjuvant therapy, Ampullary cancer

## Abstract

Periampulläre Neoplasien sind eine heterogene Gruppe verschiedener Tumorentitäten der periampullären Region, von denen das Pankreasadenokarzinom mit 60–70 % am häufigsten ist. Wie typisch für Pankreaskarzinome zeichnen sich periampulläre Pankreaskarzinome durch ein aggressives Wachstum und eine frühe systemische Progression aus. Aufgrund ihrer besonderen Lage in unmittelbarer Nähe zur Papilla Vateri treten Symptome in eher früherem Tumorstadium auf, sodass die Therapiemöglichkeiten und Prognose insgesamt günstiger sind als bei Pankreaskarzinomen anderer Lokalisation. Trotzdem unterscheiden sich die Therapieprinzipien bei periampullären Pankreaskarzinomen nicht wesentlich von den Standards bei Pankreaskarzinomen anderer Lokalisation. Ein potenziell kurativer Therapieansatz beim nichtmetastasierten periampullären Pankreaskarzinom ist multimodal und besteht aus der Durchführung einer partiellen Duodenopankreatektomie als radikale onkologische Resektion in Kombination mit einer systemischen, meist adjuvant verabreichten Chemotherapie. Bei Patienten mit günstigen prognostischen Faktoren kann hierdurch ein Langzeitüberleben erzielt werden. Zudem wurden mit der Weiterentwicklung der Chirurgie und Systemtherapie auch potenziell kurative Therapiekonzepte für fortgeschrittene, früher irresektable Tumoren etabliert, welche nun nach Durchführung einer neoadjuvanten Therapie oft einer Resektion zugeführt werden können. In diesem Beitrag werden die aktuellen chirurgischen Prinzipien der radikalen onkologischen Resektion periampullärer Pankreaskarzinome im Kontext der multimodalen Therapie dargestellt und ein Ausblick auf mögliche künftige Entwicklungen der Therapie gegeben.

## Abstract (en)

Periampullary neoplasms are a heterogeneous group of different tumor entities arising from the periampullary region, of which pancreatic ductal adenocarcinoma (PDAC) is the most common subgroup with 60–70%. As typical for pancreatic adenocarcinomas, periampullary pancreatic cancer is characterized by an aggressive growth and early systemic progression. Due to the anatomical location in close relationship to the papilla of Vater symptoms occur at an earlier stage of the disease, so that treatment options and prognosis are overall more favorable compared to pancreatic carcinomas at other locations. Nevertheless, the principles of treatment for periampullary pancreatic cancer are not substantially different from the standards for pancreatic cancer at other locations. A potentially curative approach for non-metastatic periampullary pancreatic cancer is a multimodal therapy concept, which includes partial pancreatoduodenectomy as a radical oncological resection in combination with a systemic adjuvant chemotherapy. As a result, long-term survival can be achieved in patients with favorable prognostic factors. In addition, with the continous development of surgery and systemic treatment potentially curative treatment concepts for advanced initially nonresectable tumors were also established, after completion of neoadjuvant treatment. This article presents the current surgical principles of a radical oncological resection for periampullary pancreatic cancer in the context of a multimodal treatment concept with an outlook for future developments of treatment.

## Einteilung periampullärer Tumoren

**Figure 1:**
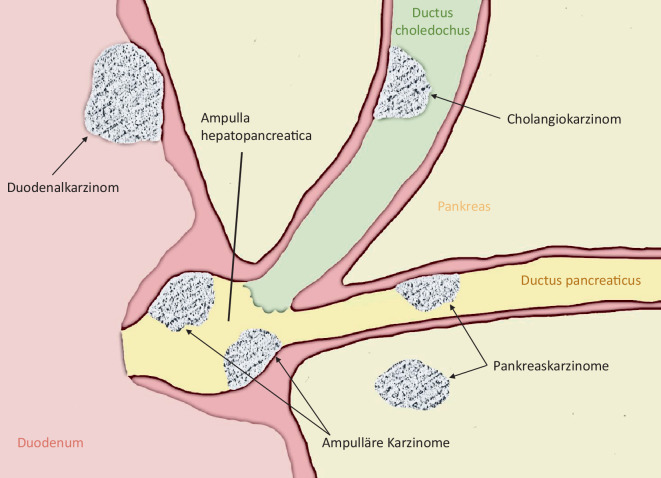

Periampulläre Tumoren liegen in einer komplexen anatomischen Region, in welcher sich in der Regel der Ductus pancreaticus major und der Ductus choledochus als Ampulla hepatopancreatica (Ampulla Vateri) vereinen und in der Papilla duodeni major (Papilla Vateri) gemeinsam ins Duodenum münden, wenn nicht angeborene Varianten vorliegen (Abb. 1). Entsprechend dieser besonderen anatomischen Lagebeziehung lassen sich verschiedene Epitheltypen unterscheiden, aus denen entsprechende Neoplasien hervorgehen können [1]. Sie umfassen folgende Entitäten:

- nichtpankreatische periampulläre Tumoren,
  - Duodenaladenome/-karzinome (10 %),
  - ampulläre Karzinome (10–20 %),
  - Cholangiokarzinome des distalen Ductus choledochus (10 %),
- pankreatische periampulläre Tumoren,
  - duktales Adenokarzinom des Pankreas (60–70 %),
  - neuroendokrine und gemischt neuroendokrine/nichtneuroendokrine Neoplasien (< 1 %) [2],
- periampulläre Adenokarzinome nichtidentifizierbaren Ursprungs (PRAIO; ~ 5 %; [3]).

Bei den periampullären Karzinomentitäten, welche keinem duktalen Adenokarzinom des Pankreas entsprechen, können intestinale oder pankreatikobiliäre Typen unterschieden werden [4]. Für die Raumforderungen mit intestinaler Ausprägung gilt, dass diese meist von der duodenalen Oberfläche der Ampulle ausgehen („[peri]ampullary-duodenal“), wohingegen Tumoren mit pankreatikobiliärer Differenzierung den tieferen Anteilen der Ampulle entstammen („ampullary-ductal“; [5]). Periampulläre Adenokarzinome des Pankreas entstammen dem papillennahen Pankreasparenchym und Gangsystem, wobei der Übergang zu nicht periampullären Pankreaskopfkarzinomen fließend ist.

Periampulläre Karzinome mit duodenalem Ursprung weisen in über 60 % eine intestinale Differenzierung auf, ampulläre Karzinome zeigen zu etwa gleichen Anteilen eine intestinale oder pankreatikobiliäre Differenzierung und distale Cholangiokarzinome haben zu über 85 % eine pankreatikobiliäre Differenzierung [6]. Die unterschiedlichen histopathologischen Typen sind prognostisch relevant. So haben Patienten mit einer intestinalen Differenzierung ein deutlich längeres medianes Überleben von 71 Monaten im Vergleich zu Patienten mit einer pankreatikobiliären Differenzierung mit einem medianen Überleben von nur 33 Monaten [6]. Ebenfalls liegen die 5‑Jahres-Überlebensraten für intestinal differenzierte Tumoren deutlich über denen für Tumoren mit pankreatikobiliärer Differenzierung (52 % vs. 29 %; [5, 7]).

Eine exakte präoperative Diagnosestellung des histologischen Typs ist schwierig

Eine exakte präoperative Diagnosestellung des histologischen Typs periampullärer Tumoren ist häufig nicht sicher zu ermitteln. So wurde in einer kürzlich veröffentlichten multizentrischen Studie an über 1200 Patienten mit periampullären Tumoren gezeigt, dass einerseits in 13 % der vermuteten Fälle eines Pankreaskarzinoms ein nichtpankreatischer periampullärer Tumor vorlag und andererseits von den vermuteten nichtpankreatischen periampullären Tumoren 21 % postoperativ als Pankreaskarzinom identifiziert wurden [8]. Eine aktuelle Studie aus Japan berichtet, dass bei über 5 % aller Patienten mit periampullären Tumoren ein nichtidentifizierbarer Ursprung vorliegt („periampullary region adenocarcinomas with an indeterminable origin“, [PRAIO]; [3]). Diese PRAIOs tragen eigenständige histopathologische Merkmale, sodass das Vorliegen einer zusätzlichen Entität periampullärer Tumoren diskutiert wird. In Zukunft sind weitere Studien notwendig, um auch auf molekularbiologischer Ebene die komplexe Heterogenität periampullärer Tumoren weiter zu entschlüsseln.

Der Schwerpunkt dieses Artikels stellt die Chirurgie des periampullären duktalen Adenokarzinoms des Pankreas dar (im Folgenden Pankreaskarzinom genannt), welches mit über 60 % den Großteil periampullärer Malignome umfasst. Primäres Ziel ist es hierbei, den derzeitigen chirurgischen Therapiestandard darzustellen und neue chirurgische Strategien unter dem zunehmenden Einfluss multimodaler Therapiekonzepte zu diskutieren. Zu den meisten Aspekten der chirurgischen und multimodalen Therapie gibt es keine spezifischen Daten für das periampulläre Pankreaskarzinom, sondern nur für das Pankreaskarzinom unabhängig von der Lokalisation im Pankreas. Prinzipiell unterscheiden sich die Therapieprinzipien und chirurgischen Standards für das periampulläre Pankreaskarzinom daher nicht von denen des nicht unmittelbar periampullären Pankreas(kopf)karzinoms.

## Onkologische Resektion und Überleben

Beim Pankreaskarzinom handelt es sich um eine aggressive Tumorentität, welche sich durch eine frühe systemische Metastasierung auszeichnet [9]. Die chirurgische Resektion in Verbindung mit einer modernen Chemotherapie bilden die Grundpfeiler der potenziell kurativen Therapie und können ein Langzeitüberleben erreichen [9]. In Bezug auf periampulläre Tumoren begünstigt zwar die Lage im proximalen Pankreas eine Diagnosestellung in früheren Tumorstadien mit entsprechend höheren Resektionsraten im Vergleich zu Tumoren im Pankreaskorpus- oder -schwanzbereich, jedoch liegen auch hier zum Zeitpunkt der chirurgischen Resektion schon häufig Mikrometastasen vor [10]. Daher ist eine sorgfältige präoperative Patientenselektion anhand anatomischer und biologischer Parameter in Verbindung mit einer radikalen chirurgischen Entfernung des Primärtumors unerlässlich, um in Kombination mit modernen (neo‑)adjuvanten Therapiekonzepten ein Langzeitüberleben zu erreichen [11, 12] (s. a. Artikel „Präoperative Diagnostik periampullärer Karzinome“ und „Multimodale Therapie periampullärer Karzinome“ in dieser Ausgabe von *Der Chirurg*).

### Kriterien der Resektabilität

Periampulläre Pankreaskarzinome lassen sich in erster Linie in Abhängigkeit ihres Lagebezuges zu den benachbarten großen Oberbauchgefäßen in verschiedene Kategorien der Resektabilität einteilen [13–15]. Neben diesen anatomischen Kriterien werden in einigen Empfehlungen auch biologische Faktoren wie die Höhe des Tumormarkers CA19‑9 („carboanhydrate antigen 19-9“) und das in der Bildgebung vermutete Vorliegen von Lymphknotenmetastasen sowie die körperliche Verfassung des Patienten (ECOG[Eastern Cooperative Oncology Group]-Status) berücksichtigt [16]. Wesentliches Ziel ist es, anhand der anatomischen Resektabilität ein sinnvolles Therapiekonzept im Sinne einer primär chirurgischen oder einer vorgeschalteten systemischen Chemotherapie festzulegen. Dabei lassen sich Pankreaskarzinome anatomisch in primär resektable, Borderline-resektable oder nichtresektable Tumoren klassifizieren (Tab. 1).

**Table 1:** 

	Lokal begrenzt	Lokal fortgeschritten (Borderline-resektabel)	Lokal fortgeschritten (Nichtresektabel)
*AHPBA/SSO/SSAT (2009; [* 14*])*			
V. portae/V. mesenterica sup	Kein Kontakt	Kontakt oder Ummauerung	Kontakt oder Ummauerung
Truncus coeliacus	Fettlamelle zum Gefäß	Kein Kontakt, Okklusion oder Ummauerung	Ummauerung
A. mesenterica sup	Fettlamelle zum Gefäß	Kontakt < 180°	Ummauerung
A. hepatica communis	Fettlamelle zum Gefäß	Kontakt/kurze Okklusion ohne TC Kontakt	Ummauerung
*NCCN-Leitlinien (2017; [* 15*])*			
V. portae/V. mesenterica sup	Kein Kontakt oder Kontakt < 180°	Kontakt > 180° oder < 180° mit Thrombus/Irregularität	Nichtrekonstruierbare Okklusion
Truncus coeliacus	Kein Kontakt	Kein Kontakt oder > 180° mit freier Aorta/AGD	Ummauerung
A. mesenterica sup	Kein Kontakt	Kontakt < 180°	Ummauerung
A. hepatica communis	Kein Kontakt	Kontakt oder kurzstreckige Okklusion	Ummauerung
*IAP-Leitlinien (2018; [* 16*])*			
V. portae/V. mesenterica sup	Kein Kontakt oder unilaterale Einengung	Kontakt > 180° oder bilaterale Einengung/Okklusion bis Unterrand Duodenum	Bilaterale Einengung/Okklusion über das Duodenum hinaus
Truncus coeliacus	Kein Kontakt	Kontakt < 180° ohne Stenose	Kontakt > 180°
A. mesenterica sup	Kein Kontakt	Kontakt < 180° ohne Stenose	Kontakt > 180°
A. hepatica communis	Kein Kontakt	Kontakt ohne Kontakt AHP/TC	Kontakt/Infiltration mit Kontakt/Infiltration AHP/TC
Biologische Faktoren	CA19‑9 ≤ 500 U/ml	CA19‑9 > 500 U/ml oder positive Lymphknoten (Biopsie/PET-CT)	**–**
Konditionelle Faktoren	ECOG-Status 0–1	ECOG-Status ≥ 2	**–**

*AHPBA* The American Hepato-Pancreato-Biliary Association, *SSO* Society of Surgical Oncology, *NCCN* The National Comprehensive Cancer Network, *IAP* International Association of Pancreatology, *AGD* A. gastroduodenalis, *AHP* A. hepatica propria, *CA19‑9* „carboanhydrate antigen“ 19‑9, *ECOG* Eastern Cooperative Oncology Group Performance Status,* PET-CT* Positronenemissionstomographie-Computertomographie, *TC* Truncus coeliacus

Für Patienten mit resektablem und Borderline-resektablem Pankreaskarzinom kann auf Basis der aktuellen Datenlage eine primäre chirurgische Therapie empfohlen werden, wobei neoadjuvante Konzepte derzeit in randomisiert-kontrollierten Studien überprüft werden [17]. Lokal fortgeschrittene Pankreaskarzinome mit Infiltration oder Ummauerung der Arteria mesenterica superior oder des Truncus coeliacus sind per definitionem nicht resektabel und sollten daher mit einer neoadjuvanten Chemotherapie behandelt werden [18]. In der Praxis erfolgen die Beurteilung der Resektabilität und die Behandlungsallokation jedoch auch und gerade in interdisziplinären Tumorboards häufig auf der Grundlage persönlicher Erfahrungen und anhand nichtstandardisierter Parameter, sodass hier eine große Behandlungsheterogenität zu verzeichnen ist [19]. Daher ist eine verlässliche prätherapeutische Prognostizierung von Patienten mit Pankreaskarzinom anhand objektiver Prognoseparametern wünschenswert, um in Zukunft objektive und an die individuelle Prognose angepasste Therapieentscheidungen treffen zu können [9]. Ein solches objektives Instrument für die prätherapeutische Prognoseeinschätzung könnte zukünftig der Heidelberg Prognostic Pancreatic Cancer Score darstellen, welcher auf objektive klinische und laborchemische Parameter zurückgreift und eine prognostisch relevante Stratifizierung von Patienten mit potenziell resektablem Pankreaskarzinom erlaubt [11].

### Präoperative Interventionen

Insbesondere bei Pankreaskopfkarzinomen kann es durch die Tumorlokalisation häufig zur Ausbildung eines Verschlussikterus kommen. Bei konsekutiver Cholangitis und Leberdysfunktion kann die präoperative endoskopische retrograde Cholangiopankreatikographie (ERCP) und Stenteinlage zur Galleableitung angezeigt sein. Für eine routinemäßige präoperative biliäre Drainage gibt es jedoch keine Empfehlung, wenn die Resektion zeitnah erfolgen kann [20]. Die präoperative Stenteinlage führt nach Resektion zu einer höheren Rate infektiöser Komplikationen im Vergleich zu Patienten mit primärer Operation ohne Stent (74 % vs. 39 %) [21, 22]. Diese Erkenntnisse treffen auch auf die Subgruppe von Patienten mit periampullären Pankreaskarzinomen zu, sodass auch bei diesen Patienten keine Empfehlung für ein routinemäßiges präoperatives Stenting ausgesprochen werden kann [23].

### Operatives Vorgehen

**Figure 2:**
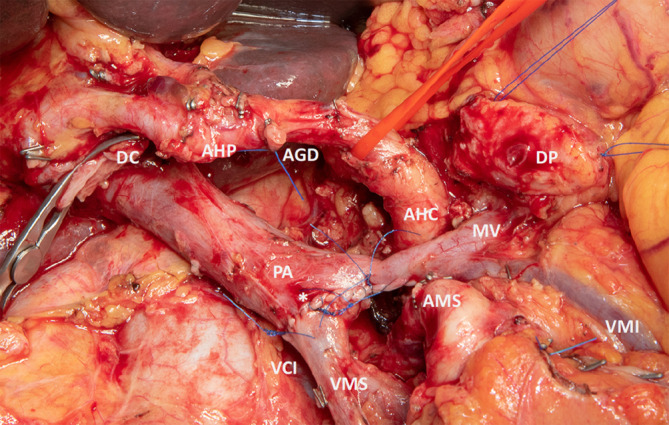

Grundsätzlich ist die radikale onkologische Resektion bei periampullären Pankreaskarzinomen die Therapie der Wahl als Grundvoraussetzung für ein kuratives Behandlungskonzept (Abb. 2; [24]). Immer wenn der Verdacht auf ein invasives Karzinom besteht, muss eine formelle onkologische Resektion im Sinne einer partiellen Duodenopankreatektomie mit systematischer Lymphadenektomie durchgeführt werden [25]. Diese Operation wird in folgenden Schritten durchgeführt:

#### Explorationsphase

Bei der konventionellen Operation sind die mediane und quere Oberbauchlaparotomie gleichwertige Zugangsmöglichkeiten [26, 27]. Nach Ausschluss von Lebermetastasen und Peritonealkarzinose wird die Exploration fortgesetzt, indem in die Bursa omentalis eingegangen und das Duodenum mittels Kocher-Manöver mobilisiert wird. Zur frühen Klärung der lokalen Resektabilität wird der Bezug des Tumors zu den arteriellen Oberbauchgefäßen im Sinne eines „artery-first approach“ überprüft [28]. Je nach bildmorphologischem Befund kann dabei derjenige Zugangsweg gewählt werden, welcher zur Klärung des Bezugs zu dem im spezifischen Fall für die Resektabilität kritischen Gefäß führt [29]. Die frühzeitige Darstellung der perivaskulären Dissektionsebenen erlaubt zudem eine kontrollierte und risikoarme Resektionsphase (vgl. unten). Aufgrund der tendenziell früheren klinischen Manifestation sind periampulläre Pankreaskarzinome seltener lokal fortgeschritten als papillenferne Pankreaskarzinome. Dennoch kann auch bei periampullären Pankreaskarzinomen eine relativ enge anatomische Lage zur A. mesenterica superior, der A. hepatica und dem Truncus coeliacus bestehen, weshalb die beschriebenen Techniken standardisiert eingesetzt werden sollten. Im Falle einer möglichen Infiltration der portomesenterischen Achse muss vor der Durchführung irreversibler Operationsschritte die venöse Rekonstruktionsmöglichkeit sichergestellt werden [30].

#### Prinzipen der Resektionsphase

In der Frühphase der Resektion wird das Ligamentum hepatoduodenale präpariert und eine Cholezystektomie mit anschließender Durchtrennung des Ductus hepatocholedochus durchgeführt. Hierbei sollte die intraoperative Schnellschnittuntersuchung des proximalen Gallengangs und insbesondere bei präoperativer Gallengangsdrainage die Entnahme von Gallenflüssigkeit für die mikrobiologische Untersuchung erfolgen, da speziell die interne Gallengangsdrainage mit einer Bakteriobilie assoziiert ist und bei postoperativen Infektionen so eine antibiogrammgerechte Antibiotikatherapie möglich wird [31, 32]. Weiterhin erfolgt in dieser Phase der Resektion das Absetzen des Magens oder des Duodenums, je nachdem ob eine pyloruserhaltende oder pylorusresezierende Operation durchgeführt wird. Nach derzeitiger Datenlage sind beide Verfahren insbesondere hinsichtlich des Auftretens einer postoperativen Magenentleerungsstörung gleichwertig [33]. Jedoch kann aus onkologischen Gründen die Resektion des Pylorus oder distalen Magens erforderlich sein oder aufgrund von Perfusions- oder Abflussstörungen notwendig werden. Insbesondere bei Eingriffserweiterungen im Sinne einer totalen Pankreatektomie kann die insuffiziente venöse Drainage des Magens zu schwerwiegenden Komplikationen führen [34].

Bei periampullären Karzinomen ist die radikale Resektion die einzige kurative Behandlungsoption

Die Präparation des Ligamentum hepatoduodenale wird entlang der A. hepatica propria bis zum Abgang der A. gastroduodenalis fortgesetzt. Letztere wird vor ihrer Durchtrennung zur Probe geklemmt, um eine erhaltene Leberdurchblutung über den Truncus coeliacus zu sichern. Zeigt die präoperative Diagnostik bereits ein komprimierendes Ligamentum arcuatum, kann es erforderlich sein, dieses freizulegen und zu spalten. Anschließend wird die A. gastroduodenalis abgesetzt, wodurch die suprapankreatische Pfortader zur Darstellung kommt. Entlang dieser Leitstruktur wird das Pankreas unterfahren, um dieses im weiteren Verlauf linksseitig des Tumors zu durchtrennen. Eine Schnellschnittuntersuchung des Pankreasabsetzungsrandes ist obligat und erfordert im Fall von Tumorzellen im Absetzungsrand eine Nachresektion des Pankreas. Die Resektion wird fortgesetzt, indem die erste Jejunalschlinge distal des Treitz-Bandes durchtrennt wird. Das proximale Ende wird bis zum Treitz-Band skelettiert und unter dem Treitz-Band hindurchgezogen. Die Mobilisation des Pankreaskopfes beginnt mit der Dissektion des Mesopankreas von der Adventitia der V. und A. mesenterica superior (Dissektionslevel 3 nach Inoue und Kollegen) vom Processus uncinatus beginnend in kaudokranialer Richtung unter sicherer Schonung der Gefäße [30]. Eine so an den Gefäßen orientierte Resektionstechnik resultiert immer auch in der Mitnahme der regionalen Lymphknoten im Sinne einer adäquaten und systematischen regionalen Lymphadenektomie, welche im folgenden Abschnitt genauer erläutert wird [35, 36].

##### Systematische Lymphadenektomie.

Die onkologische Resektion beinhaltet eine standardisierte Lymphadenektomie, welche nach den Empfehlungen der International Study Group of Pancreatic Surgery (ISGPS) in Anpassung an die japanische Lymphknotenklassifizierung erfolgen sollte [36]. Diese beinhaltet eine komplette peripankreatische Lymphadenektomie im Bereich des Pankreaskopfes (Stationen 13, 17) und des Pylorus (5, 6) sowie eine Lymphadenektomie im Bereich des Ligamentum hepatoduodenale (8, 12) und entlang der V. portae, A. hepatica communis bis hin zur rechten Seite des Truncus coeliacus (9). Weiterhin gilt es, die Lymphknoten an der rechten Seite der A. und V. mesenterica superior komplett zu entfernen (14). Wesentliches Ziel der systematischen Lymphadenektomie ist es, alle positiven Lymphknoten zu entfernen, welche entscheidend für das Tumorstadium und die Prognose des Patienten sind [35, 37].

Eine adäquate Lymphadenektomie bei periampullären Karzinomen verbessert die Prognose

Als Qualitätsmaß für eine sorgfältige Lymphadenektomie wird von der ISGPS eine Mindestanzahl von 15 zu entfernenden Lymphknoten empfohlen, wobei die Anzahl an zu untersuchenden Lymphknoten auch von der Qualität der pathologischen Begutachtung abhängt [36]. Eine in Heidelberg durchgeführte Studie mit systematischer Lymphadenektomie analog der ISGPS-Empfehlungen ergab eine mediane Anzahl von 24 untersuchten Lymphknoten in Duodenopankreatektomiepräparaten von 811 Patienten mit Pankreaskarzinom [35]. Für eine erweiterte Lymphknotendissektion unter Einschluss der extraregionalen (parakaval/interaortokaval/paraaortal) Lymphknotenstationen konnte in mehreren Studien kein Überlebensvorteil bei gleichzeitigem Anstieg der Morbidität gezeigt werden, sodass diese nicht als Standardprozedur erfolgen sollte [30, 38].

##### R-Status.

Seit Mitte der 2000er-Jahre wird in Europa basierend auf Arbeiten aus Heidelberg und Leeds eine standardisierte pathologische Aufarbeitung und strikte Definition des R‑Status für Pankreaskarzinome angewendet [39]. Dieser Standard sieht bereits eine R1-Situation vor, wenn Tumorzellen bis zu 1 mm an den peripankreatischen Schnittrand heranreichen. Dies gilt sowohl für alle zirkumferenziellen Ränder als auch für die Absetzungsränder am Präparat. Zahlreiche Studien haben diese strikte Definition untersucht, mit teils sehr inhomogenen R0-Resektionsraten und daraus hervorgehenden Überlebenszahlen [40]. In einer großen Studie zu Pankreaskopfresektionen, bei denen auch periampulläre Karzinome eingeschlossen wurden, konnte gezeigt werden, dass Patienten mit einer direkten R1-Situation die schlechteste Prognose hatten, im Gegensatz zu Patienten mit R1 (< 1 mm) oder einer strikten R0-Resektion mit einem medianem Gesamtüberleben von 23 vs. 27 vs. 41 Monaten [41]. Diese Ergebnisse konnten in einer Validierungsstudie auch für andere Tumorlokalisationen des Pankreas bestätigt werden [42].

In den aktuellen S3-Leitlinien wird der strikte R‑Status analog zum Rektumkarzinom anhand des „Konzeptes des zirkumferenziellen Resektionsrands (CRM)“ erfasst. Dies beinhaltet die Klassifikation als CRM^+^ (R0 „narrow“) beim Vorliegen von Tumorzellen innerhalb des 1‑mm-Sicherheitsabstands bzw. als CRM^–^ (R0 „wide“) bei tumorfreien Präparaterändern [20]. Um die prognostische Aussagekraft des R‑Status künftig noch besser erfassen und analysieren zu können, ist die genaue Angabe der Distanz des Tumors zum nächstgelegenen Präparaterand sowie dessen Lokalisation von besonderer Bedeutung.

##### Gefäßresektionen.

In den vergangenen Jahren sind die chirurgischen Techniken für Pankreasresektionen weiter verfeinert und die Indikationen für erweiterte Resektionen einschließlich Gefäßresektionen ausgedehnt worden, insbesondere auch vor dem Hintergrund einer zunehmenden Population an neoadjuvant behandelten Patienten [30]. Zwar ist das Überleben nach erweiterten Resektionen aufgrund des fortgeschrittenen Tumorstadiums im Vergleich zu Standardresektionen meist schlechter, jedoch übersteigen die onkologischen Ergebnisse erweiterter Resektionen die Ergebnisse einer palliativen Therapie [43]. Die größte monozentrische Studie zu erweiterten Resektionen mit insgesamt 1635 Patienten demonstrierte ein medianes Überleben von 16,1 Monaten mit einer 5‑Jahres-Überlebensrate von 11 % in der Subgruppe von Patienten mit erweiterter Resektion im Vergleich zu Patienten mit Standardresektion, welche ein medianes Überleben von 23,6 Monaten hatten und eine 5‑Jahres-Überlebensrate von 20,6 % [44].

In Bezug auf venöse Resektionen (s. Abb. 2) konnte in einer großen Metaanalyse mit über 2200 Patienten gezeigt werden, dass die 1‑, 3‑ und 5‑Jahres-Überlebensraten bei Patienten mit venöser Resektion vergleichbar waren zu Patienten, bei denen keine Gefäßresektion notwendig war (61,3, 19,4 und 12,3 % vs. 61,8, 26,6 und 17 %). Zwar waren der Blutverlust und die Dauer der Operation erhöht bei Patienten mit venöser Resektion, jedoch konnte kein Unterschied in Bezug auf die Morbidität (Odds Ratio: 0,95; 95 %-Konfidenzintervall: 0,74–1,21; *p* = 0,67) und Mortalität (Gefäßresektion 3,3 % vs. Standardresektion 3,7 %) zwischen den Gruppen festgestellt werden [45]. In einer multizentrischen Studie aus Japan an 937 Patienten mit Pankreaskopfresektionen wurden bei 435 (46,4 %) Gefäßresektionen durchgeführt. Auch hier waren die Morbidität und Mortalität zwischen den Gruppen vergleichbar. Das mediane Gesamtüberleben lag nach venöser Resektion bei 18,5 Monaten im Vergleich zu 25,8 Monaten bei Patienten ohne Gefäßresektion, wobei die Tumoren in der Gruppe mit Gefäßresektionen fortgeschrittener waren [46].

Eine genaue Angabe des Resektionsstatus von periampullären Karzinomen dient der Qualitätssicherung

Im Gegensatz zu venösen Resektionen gehen arterielle Resektionen mit einer signifikant höheren Morbiditäts- und Mortalitätsrate einher, sodass bei arterieller Infiltration im Allgemeinen keine primäre Resektion empfohlen werden kann [30]. Kürzlich konnte jedoch gezeigt werden, dass ein arterielles „divestment“ mit longitudinaler Abtragung des periarteriellen Nervenplexus oder der Adventitia bei Patienten mit vorausgehender neoadjuvanter Therapie sicher durchzuführen ist und dadurch etwaige residuelle Tumorzellen ohne arterielle Resektion entfernt werden können [47]. Aus dieser Erfahrung heraus wurde ferner das „Triangle-Prinzip“ für neoadjuvant behandelte Patienten konzipiert [48]. Dabei wird ebenfalls ein arterielles „divestment“ durchgeführt, um das fibrotische Bindegewebe im Dreieck zwischen A. hepatica communis, A. mesenterica superior und V. portae zu entfernen [49]. Die onkologischen Ergebnisse dieser neuen radikalen Techniken sind noch unbekannt und Gegenstand aktueller Studien.

#### Rekonstruktionstechniken

Eine wichtige Fragestellung in der Pankreaschirurgie ist der wissenschaftliche Vergleich der alternativen Rekonstruktiontechniken in Bezug auf die resultierenden postoperativen Ergebnisse. Wichtigstes Ziel ist die Vermeidung der postoperativen Pankreasfistel (POPF) als zentrale Komplikation nach Pankreasresektionen. Die POPF wurde daher meist als wichtigster Endpunkt entsprechender randomisiert-kontrollierter Studien gewählt [50–52]. Basierend auf der verfügbaren Evidenz sollte die Rekonstruktionsphase möglichst standardisiert erfolgen und im Bedarfsfall der klinischen Situation angepasst werden. Die Pankreasanastomose lässt sich grundsätzlich mittels Pankreatikogastrostomie oder Pankreatikojejunostomie durchführen, wobei bislang kein eindeutiger Vorteil des einen oder anderen Verfahrens bewiesen werden konnte; insbesondere ist das Risiko für das Auftreten einer POPF vergleichbar [53]. Für die Durchführung der Pankreatikojejunostomie hat sich in unserer Erfahrung eine zweireihige End-zu-Seit-Pankreatojejunostomie (Bern/Heidelberg Technik; [54, 55]) im Gegensatz zur klassischen Blumgart-Anastomose bewährt, wobei es auch hierfür keine eindeutige Evidenz und dementsprechend keine abschließende Empfehlung durch die ISGPS gibt [56].

Die Anastomosierung von Restpankreas, Gallengang und Magen/Duodenum an dieselbe Schlinge ist aufgrund der relativ einfachen Technik und basierend auf der aktuellen Datenlage als Standardrekonstruktion anzusehen [56, 57]. So bringt die Durchführung von Pankreas- und Gallengangsanastomose in getrennt ausgeschalteten Schlingen keinen nachweisbaren Nutzen und ist aufgrund des größeren technischen und zeitlichen Aufwandes nicht zu empfehlen [58]. Weiterhin war die Separation des Magens von Pankreas- und Gallengangsanastomose in einer randomisiert-kontrollierten Studie ohne Vorteil [59]. Für die Rekonstruktion des alimentären Traktes konnte in einer weiteren kürzlich publizierten Studie gezeigt werden, dass die retrokolische und antekolische Rekonstruktion in Bezug auf die postoperative Magenentleerungsstörung gleichwertig sind [60]. Eine aktuelle Netzwerkmetaanalyse empfiehlt die Durchführung einer antekolischen Rekonstruktion mit Braun’scher-Fußpunktanastomose als Billroth-II-Rekonstruktion nach vorausgehender Resektion des Pylorus [61], wobei wie oben ausgeführt die Reduktion der Magenentleerungsstörung durch Pylorusresektion nicht bewiesen ist [33].

## Rolle der minimal-invasiven Chirurgie

Die Rolle der minimal-invasiven Chirurgie für Pankreaskopfresektionen ist aktuell ein wichtiges Thema und wird in zahlreichen Studien untersucht. Aufgrund der Komplexität insbesondere der Rekonstruktion ist ein laparoskopisches oder robotisches Vorgehen bei der partiellen Duodenopankreatektomie deutlich anspruchsvoller als bei distalen Resektionen, welche bereits oft routinemäßig minimal-invasiv durchgeführt werden [62]. Bislang konnten randomisiert-kontrollierte Studien zur offenen vs. laparoskopischen Duodenopankreatektomie, welche einem hohen Selektionsbias ausgesetzt sind, nur einen marginalen Vorteil in Bezug auf die Krankenhausverweildauer (–1 Tag) und den intraoperativen Blutverlust (–150 ml) im Vergleich zur konventionellen Chirurgie aufweisen [63, 64]. Demgegenüber stehen die deutlich längere Operationsdauer (+95 min) sowie eine sehr lange Lernkurve, für die es eine große Anzahl von Prozeduren bedarf (> 100 Standardresektionen; [63]). Darüber hinaus wurde die niederländische LEOPARD-2-Studie aufgrund eines 5‑fach erhöhten komplikationsassoziierten Mortalitätsrisikos in der laparoskopischen Gruppe abgebrochen, obwohl die Operation von erfahrenen minimal-invasiven Chirurgen durchgeführt wurde [65].

Die Robotik bietet im Gegensatz zur konventionellen Laparoskopie den großen Vorteil der dreidimensionalen Handhabung der Arbeitsinstrumente, was für eine sorgfältige und radikale onkologische Resektion, aber auch für die Rekonstruktion enorme Vorteile hat. Veröffentlichungen zur robotischen Duodenopankreatektomie nehmen seit ihrer Erstbeschreibung im Jahr 2003 stetig zu. Doch fehlen nach wie vor Ergebnisse aus randomisiert-kontrollierten Studien [66]. Eine große retrospektive multizentrische Studie aus den USA zum Vergleich der konventionellen mit der robotischen Pankreatoduodenektomie mit über 1000 Patienten in acht teilnehmenden Zentren zeigt gute Ergebnisse, wobei die Behandlungsgruppen aufgrund von Selektionsbias nur eingeschränkt vergleichbar sind [67]. Die Vor- und Nachteile der robotischen Chirurgie müssen insbesondere in Bezug auf die postoperative Morbidität und das onkologische Outcome, aber auch unter ökonomischen Gesichtspunkten in prospektiven Studien weiter untersucht werden. Für eine gute Operationsqualität und vergleichbare Ergebnisse ist jedenfalls ein standardisiertes Vorgehen und Trainingsprogramm hier besonders wichtig [68].

## Postoperative Komplikationen und Patientensicherheit

Die Ergebnisse der chirurgischen Therapie von Pankreaskarzinomen haben sich in den letzten beiden Jahrzehnten deutlich verbessert, wobei insbesondere die operationsassoziierte Mortalität, aber auch Morbidität durch verschiedene Faktoren deutlich reduziert werden konnten [9]. Dazu zählt vor allem ein verbessertes interdisziplinäres Management postoperativer Komplikationen mit wesentlichen Fortschritten in der Intensivmedizin und einem zunehmenden Stellenwert der interventionellen Radiologie.

Auch nach Resektionen periampullärer Karzinome ist die Pankreasfistel die wichtigste Komplikation

Nach wie vor stellt die POPF mit ca. 20–25 % eine häufige und schwerwiegende Komplikation nach Pankreaskopfresektionen dar [69]. Die Häufigkeit von Pankreasfisteln konnte trotz zahlreicher Untersuchungen verschiedener Anastomosentechniken inklusive Pankreasgangableitungen nicht signifikant reduziert werden [53, 56]. Auch für den systemischen Einsatz von Somatostatinanaloga oder die lokale Applikation von Fibrinklebern konnte in entsprechenden Studien keine Reduktion der POPF-Rate nachgewiesen werden [70, 71]. Daher besteht die aktuelle Empfehlung, dass bei Risikoanastomosen der erfahrenere Chirurg die Anastomose anlegen sollte, da das POPF-Risiko zwar nicht durch eine spezielle Technik verringert werden kann, jedoch erfahrenere Chirurgen sehr wohl bessere Ergebnisse haben. Darüber hinaus können zur (intraoperativen) Risikostratifizierung validierte klinische Scores zur Anwendung kommen, die das individuelle POPF-Risiko ermitteln [72, 73].

Zur Patientensicherheit trägt weiterhin eine Zentrumsbildung in der Pankreaschirurgie bei. Zwei Analysen von Routinedaten zeigten in diesem Zusammenhang einen klaren Bezug von Fallzahl und (perioperativer) Mortalität nach Pankreaschirurgie in Deutschland [74, 75]. So konnte eine risikoadaptierte Reduktion der postoperativen Mortalität zwischen Zentren mit sehr hohen Fallzahlen und Zentren mit sehr niedrigen Fallzahlen demonstriert werden (6,5 % vs. 11,5 %; OR 0,47; [75]). Bei nach wie vor hoher Morbidität nach Pankreaschirurgie lässt sich dieser Zusammenhang mit einem routinierteren und effektiveren Management schwerwiegender Komplikationen in Zentren begründen [76]. Die Chirurgie periampullärer Pankreaskarzinome sollte daher in entsprechenden Zentren für Pankreaschirurgie erfolgen.

## Prognose

Insgesamt hat sich die Prognose bei resektablen Pankreaskarzinomen innerhalb der letzten beiden Jahrzehnte durch multimodale Therapiekonzepte deutlich verbessert, wobei die beobachteten 5‑Jahres-Überlebensraten nach erfolgreicher Resektion und adjuvanter Chemotherapie annähernd 20 % erreichen [77]. Diese Ergebnisse können durch das Vorliegen weiterer prognostisch relevanter Faktoren, wie ein eindeutiger R0-Status bzw. eine qualitativ hochwertige Lymphadenektomie (≥ 15 Lymphknoten), verdoppelt werden und überschreiten 50 %, wenn ausschließlich günstige prognostische Parameter vorliegen [77]. Durch den vermehrten Einsatz moderner Kombinationstherapien können diese Überlebenszahlen künftig noch weiter gesteigert werden [78, 79]. Darüber hinaus werden in Zukunft objektive klinische und biologische Parameter zur individualisierten Behandlung bei Patienten mit periampullärem Pankreaskarzinomen einen zunehmenden Stellenwert erhalten und dadurch eine individuelle Auswahl und Sequenzierung der multimodalen onkologischen Therapie im Zeitalter der personalisierten Medizin erlauben.

## Fazit für die Praxis

- Das periampulläre Pankreaskarzinom ist der häufigste Vertreter periampullärer Tumoren, bei denen eine exakte präoperative Diagnosestellung häufig erschwert ist.
- Grundsätzlich ist festzuhalten, dass sich die Therapieprinzipien und chirurgischen Standards bei periampullären Pankreaskarzinomen nicht von denen des ampullenfernen Pankreaskopfkarzinoms unterscheiden.
- Diese umfassen je nach lokalem Resektionsstatus die radikale chirurgische Duodenopankreatektomie mit systematischer Lymphadenektomie und bei Bedarf ein erweitertes Resektionsausmaß in Verbindung mit einer systemischen Chemotherapie.

## References

[1] Sarmiento JM, Nagomey DM, Sarr MG, Farnell MB (2001). Periampullary cancers: are there differences?. Surg Clin North Am.

[2] Niessen A, Schimmack S, Weber TF, Mayer P, Bergmann F, Hinz U (2021). Presentation and outcome of mixed neuroendocrine non-neuroendocrine neoplasms of the pancreas. Pancreatology.

[3] Komine R, Kojima M, Ishi G, Kudo M, Sugimoto M, Kobayashi S (2021). Recognition and pathological features of periampullary region adenocarcinoma with an indeterminable origin. Cancer Med.

[4] Westgaard A, Pomianowska E, Clausen OP, Gladhaug IP (2013). Intestinal-type and pancreatobiliary-type adenocarcinomas: how does ampullary carcinoma differ from other periampullary malignancies?. Ann Surg Oncol.

[5] Adsay V, Ohike N, Tajiri T, Kim GE, Krasinskas A, Balci S (2012). Ampullary region carcinomas: definition and site specific classification with delineation of four clinicopathologically and prognostically distinct subsets in an analysis of 249 cases. Am J Surg Pathol.

[6] Williams JL, Chan CK, Toste PA, Elliott IA, Vasquez CR, Sunjaya DB (2017). Association of histopathologic phenotype of periampullary adenocarcinomas with survival. JAMA Surg.

[7] Onkendi EO, Boostrom SY, Sarr MG, Farnell MB, Nagorney DM, Donohue JH (2012). 15-year experience with surgical treatment of duodenal carcinoma: a comparison of periampullary and extra-ampullary duodenal carcinomas. J Gastrointest Surg.

[8] van Roessel S, Soer EC, Daamen LA, van Dalen D, Farina Sarasqueta A, Stommel MWJ (2021). Preoperative misdiagnosis of pancreatic and periampullary cancer in patients undergoing pancreatoduodenectomy: a multicentre retrospective cohort study. Eur J Surg Oncol.

[9] Strobel O, Neoptolemos J, Jager D, Buchler MW (2019). Optimizing the outcomes of pancreatic cancer surgery. Nat Rev Clin Oncol.

[10] van Erning FN, Mackay TM, van der Geest LGM, Groot Koerkamp B, van Laarhoven HWM, Bonsing BA (2018). Association of the location of pancreatic ductal adenocarcinoma (head, body, tail) with tumor stage, treatment, and survival: a population-based analysis. Acta Oncol.

[11] Hank T, Hinz U, Reiner T, Malleo G, Konig AK, Maggino L (2021). A pretreatment prognostic score to stratify survival in pancreatic cancer. Ann Surg.

[12] Hank T, Sandini M, Ferrone CR, Ryan DP, Mino-Kenudson M, Qadan M (2020). A combination of biochemical and pathological parameters improves prediction of postresection survival after preoperative chemotherapy in pancreatic cancer: the PANAMA-score. Ann Surg.

[13] Bockhorn M, Uzunoglu FG, Adham M, Imrie C, Milicevic M, Sandberg AA (2014). Borderline resectable pancreatic cancer: a consensus statement by the international study group of pancreatic surgery (ISGPS). Surgery.

[14] Callery MP, Chang KJ, Fishman EK, Talamonti MS, William Traverso L, Linehan DC (2009). Pretreatment assessment of resectable and borderline resectable pancreatic cancer: expert consensus statement. Ann Surg Oncol.

[15] Tempero MA, Malafa MP, Al-Hawary M, Asbun H, Bain A, Behrman SW (2017). Pancreatic adenocarcinoma, version 2.2017, NCCN clinical practice guidelines in oncology. J Natl Compr Canc Netw.

[16] Isaji S, Mizuno S, Windsor JA, Bassi C, Fernandez-Del Castillo C, Hackert T (2018). International consensus on definition and criteria of borderline resectable pancreatic ductal adenocarcinoma 2017. Pancreatology.

[17] Klaiber U, Leonhardt CS, Strobel O, Tjaden C, Hackert T, Neoptolemos JP (2018). Neoadjuvant and adjuvant chemotherapy in pancreatic cancer. Langenbecks Arch Surg.

[18] Hank T, Strobel O (2019). Conversion surgery for advanced pancreatic cancer. J Clin Med.

[19] Kirkegard J, Aahlin EK, Al-Saiddi M, Bratlie SO, Coolsen M, de Haas RJ (2019). Multicentre study of multidisciplinary team assessment of pancreatic cancer resectability and treatment allocation. Br J Surg.

[20] Seufferlein T, Porzner M, Becker T, Budach V, Ceyhan G, Esposito I (2013). S3-guideline exocrine pancreatic cancer. Z Gastroenterol.

[21] Schwarz RE (2002). Technical considerations to maintain a low frequency of postoperative biliary stent-associated infections. J Hepatobiliary Pancreat Surg.

[22] van der Gaag NA, Rauws EA, van Eijck CH, Bruno MJ, van der Harst E, Kubben FJ (2010). Preoperative biliary drainage for cancer of the head of the pancreas. N Engl J Med.

[23] Gholami S, Brennan MF (2018). Preoperative stenting for benign and malignant periampullary diseases: unnecessary if not harmful. Surg Clin North Am.

[24] Michalski CW, Liu B, Heckler M, Roth S, Sun H, Heger U (2019). Underutilization of surgery in periampullary cancer treatment. J Gastrointest Surg.

[25] Schneider M, Hackert T, Strobel O, Buchler MW (2021). Technical advances in surgery for pancreatic cancer. Br J Surg.

[26] Seiler CM, Deckert A, Diener MK, Knaebel HP, Weigand MA, Victor N (2009). Midline versus transverse incision in major abdominal surgery: a randomized, double-blind equivalence trial (POVATI: ISRCTN60734227). Ann Surg.

[27] Brown SR, Goodfellow PB (2005). Transverse verses midline incisions for abdominal surgery. Cochrane Database Syst Rev.

[28] Weitz J, Rahbari N, Koch M, Buchler MW (2010). The „artery first“ approach for resection of pancreatic head cancer. J Am Coll Surg.

[29] Sanjay P, Takaori K, Govil S, Shrikhande SV, Windsor JA (2012). „Artery-first“ approaches to pancreatoduodenectomy. Br J Surg.

[30] Niesen W, Hank T, Buchler M, Strobel O (2019). Local radicality and survival outcome of pancreatic cancer surgery. Ann Gastroenterol Surg.

[31] Sandini M, Honselmann KC, Cereda M, Angrisani M, Gavazzi F, Wellner U (2020). The relative role of bile bacterial isolation on outcome in stent-bearing patients undergoing pancreatoduodenectomy. J Gastrointest Surg.

[32] Weniger M, Hank T, Qadan M, Ciprani D, Michelakos T, Niess H (2020). Influence of Klebsiella pneumoniae and quinolone treatment on prognosis in patients with pancreatic cancer. Br J Surg.

[33] Klaiber U, Probst P, Strobel O, Michalski CW, Dorr-Harim C, Diener MK (2018). Meta-analysis of delayed gastric emptying after pylorus-preserving versus pylorus-resecting pancreatoduodenectomy. Br J Surg.

[34] Loos M, Mehrabi A, Ramouz A, Contin P, Strobel O, Muller-Stich BP (2021). Gastric venous congestion after total pancreatectomy is frequent and dangerous. Ann Surg.

[35] Strobel O, Hinz U, Gluth A, Hank T, Hackert T, Bergmann F (2015). Pancreatic adenocarcinoma: number of positive nodes allows to distinguish several N categories. Ann Surg.

[36] Tol JA, Gouma DJ, Bassi C, Dervenis C, Montorsi M, Adham M (2014). Definition of a standard lymphadenectomy in surgery for pancreatic ductal adenocarcinoma: a consensus statement by the international study group on pancreatic surgery (ISGPS). Surgery.

[37] Tarantino I, Warschkow R, Hackert T, Schmied BM, Buchler MW, Strobel O (2017). Staging of pancreatic cancer based on the number of positive lymph nodes. Br J Surg.

[38] Dasari BV, Pasquali S, Vohra RS, Smith AM, Taylor MA, Sutcliffe RP (2015). Extended versus standard lymphadenectomy for pancreatic head cancer: meta-analysis of randomized controlled trials. J Gastrointest Surg.

[39] Esposito I, Kleeff J, Bergmann F, Reiser C, Herpel E, Friess H (2008). Most pancreatic cancer resections are R1 resections. Ann Surg Oncol.

[40] Chandrasegaram MD, Goldstein D, Simes J, Gebski V, Kench JG, Gill AJ (2015). Meta-analysis of radical resection rates and margin assessment in pancreatic cancer. Br J Surg.

[41] Strobel O, Hank T, Hinz U, Bergmann F, Schneider L, Springfeld C (2017). Pancreatic cancer surgery: the new R-status counts. Ann Surg.

[42] Hank T, Hinz U, Tarantino I, Kaiser J, Niesen W, Bergmann F (2018). Validation of at least 1 mm as cut-off for resection margins for pancreatic adenocarcinoma of the body and tail. Br J Surg.

[43] Hartwig W, Vollmer CM, Fingerhut A, Yeo CJ, Neoptolemos JP, Adham M (2014). Extended pancreatectomy in pancreatic ductal adenocarcinoma: definition and consensus of the international study group for pancreatic surgery (ISGPS). Surgery.

[44] Hartwig W, Gluth A, Hinz U, Koliogiannis D, Strobel O, Hackert T (2016). Outcomes after extended pancreatectomy in patients with borderline resectable and locally advanced pancreatic cancer. Br J Surg.

[45] Zhou Y, Zhang Z, Liu Y, Li B, Xu D (2012). Pancreatectomy combined with superior mesenteric vein-portal vein resection for pancreatic cancer: a meta-analysis. World J Surg.

[46] Murakami Y, Satoi S, Motoi F, Sho M, Kawai M, Matsumoto I (2015). Portal or superior mesenteric vein resection in pancreatoduodenectomy for pancreatic head carcinoma. Br J Surg.

[47] Loos M, Kester T, Klaiber U, Mihaljevic AL, Mehrabi A, Muller-Stich BM (2020). Arterial resection in pancreatic cancer surgery: effective after a learning curve. Ann Surg.

[48] Hackert T, Strobel O, Michalski CW, Mihaljevic AL, Mehrabi A, Muller-Stich B (2017). The TRIANGLE operation—radical surgery after neoadjuvant treatment for advanced pancreatic cancer: a single arm observational study. HPB.

[49] Diener MK, Mihaljevic AL, Strobel O, Loos M, Schmidt T, Schneider M (2021). Periarterial divestment in pancreatic cancer surgery. Surgery.

[50] Tani M, Kawai M, Hirono S, Okada KI, Miyazawa M, Shimizu A (2014). Randomized clinical trial of isolated Roux-en-Y versus conventional reconstruction after pancreaticoduodenectomy. Br J Surg.

[51] Keck T, Wellner UF, Bahra M, Klein F, Sick O, Niedergethmann M (2016). Pancreatogastrostomy versus pancreatojejunostomy for RECOnstruction after PANCreatoduodenectomy (RECOPANC, DRKS 00000767): perioperative and long-term results of a multicenter randomized controlled trial. Ann Surg.

[52] Diener MK, Seiler CM, Rossion I, Kleeff J, Glanemann M, Butturini G (2011). Efficacy of stapler versus hand-sewn closure after distal pancreatectomy (DISPACT): a randomised, controlled multicentre trial. Lancet.

[53] Cheng Y, Briarava M, Lai M, Wang X, Tu B, Cheng N (2017). Pancreaticojejunostomy versus pancreaticogastrostomy reconstruction for the prevention of postoperative pancreatic fistula following pancreaticoduodenectomy. Cochrane Database Syst Rev.

[54] Shrikhande SV, Kleeff J, Buchler MW, Friess H (2007). Pancreatic anastomosis after pancreaticoduodenectomy: how we do it. Indian J Surg.

[55] Z’Graggen K, Uhl W, Friess H, Buchler MW (2002). How to do a safe pancreatic anastomosis. J Hepatobiliary Pancreat Surg.

[56] Shrikhande SV, Sivasanker M, Vollmer CM, Friess H, Besselink MG, Fingerhut A (2017). Pancreatic anastomosis after pancreatoduodenectomy: a position statement by the international study group of pancreatic surgery (ISGPS). Surgery.

[57] Lyu Y, Wang B, Cheng Y, Xu Y, Du WB (2020). Comparison of surgical outcomes between isolated pancreaticojejunostomy, isolated gastrojejunostomy, and conventional pancreaticojejunostomy after pancreaticoduodenectomy: a systematic review and meta-analysis. BMC Gastroenterol.

[58] Klaiber U, Probst P, Knebel P, Contin P, Diener MK, Buchler MW (2015). Meta-analysis of complication rates for single-loop versus dual-loop (Roux-en-Y) with isolated pancreaticojejunostomy reconstruction after pancreaticoduodenectomy. Br J Surg.

[59] Busquets J, Martin S, Fabregat J, Secanella L, Pelaez N, Ramos E (2019). Randomized trial of two types of gastrojejunostomy after pancreatoduodenectomy and risk of delayed gastric emptying (PAUDA trial). Br J Surg.

[60] Toyama H, Matsumoto I, Mizumoto T, Fujita H, Tsuchida S, Kanbara Y (2020). Influence of the retrocolic versus antecolic route for alimentary tract reconstruction on delayed gastric emptying after pancreatoduodenectomy: a multicenter, noninferiority randomized controlled trial. Ann Surg.

[61] Varghese C, Bhat S, Wang TH, O’Grady G, Pandanaboyana S (2021). Impact of gastric resection and enteric anastomotic configuration on delayed gastric emptying after pancreaticoduodenectomy: a network meta-analysis of randomized trials. BJS Open.

[62] van Hilst J, de Rooij T, Klompmaker S, Rawashdeh M, Aleotti F, Al-Sarireh B (2019). Minimally invasive versus open distal pancreatectomy for ductal adenocarcinoma (DIPLOMA): a pan-European propensity score matched study. Ann Surg.

[63] Nickel F, Haney CM, Kowalewski KF, Probst P, Limen EF, Kalkum E (2020). Laparoscopic versus open pancreaticoduodenectomy: a systematic review and meta-analysis of randomized controlled trials. Ann Surg.

[64] Wang M, Li D, Chen R, Huang X, Li J, Liu Y (2021). Laparoscopic versus open pancreatoduodenectomy for pancreatic or periampullary tumours: a multicentre, open-label, randomised controlled trial. Lancet Gastroenterol Hepatol.

[65] van Hilst J, de Rooij T, Bosscha K, Brinkman DJ, van Dieren S, Dijkgraaf MG (2019). Laparoscopic versus open pancreatoduodenectomy for pancreatic or periampullary tumours (LEOPARD-2): a multicentre, patient-blinded, randomised controlled phase 2/3 trial. Lancet Gastroenterol Hepatol.

[66] Giulianotti PC, Coratti A, Angelini M, Sbrana F, Cecconi S, Balestracci T (2003). Robotics in general surgery: personal experience in a large community hospital. Arch Surg.

[67] Zureikat AH, Postlewait LM, Liu Y, Gillespie TW, Weber SM, Abbott DE (2016). A multi-institutional comparison of perioperative outcomes of robotic and open pancreaticoduodenectomy. Ann Surg.

[68] Zwart MJW, Nota CLM, de Rooij T, van Hilst J, Te Riele WW, van Santvoort HC (2021). Outcomes of a multicenter training program in robotic pancreatoduodenectomy (LAELAPS-3). Ann Surg.

[69] Bassi C, Marchegiani G, Dervenis C, Sarr M, Abu Hilal M, Adham M (2017). The 2016 update of the international study group (ISGPS) definition and grading of postoperative pancreatic fistula: 11 years after. Surgery.

[70] Elliott IA, Dann AM, Ghukasyan R, Damato L, Girgis MD, King JC (2018). Pasireotide does not prevent postoperative pancreatic fistula: a prospective study. HPB (Oxford).

[71] Schindl M, Fugger R, Gotzinger P, Langle F, Zitt M, Stattner S (2018). Randomized clinical trial of the effect of a fibrin sealant patch on pancreatic fistula formation after pancreatoduodenectomy. Br J Surg.

[72] Callery MP, Pratt WB, Kent TS, Chaikof EL, Vollmer CM (2013). A prospectively validated clinical risk score accurately predicts pancreatic fistula after pancreatoduodenectomy. J Am Coll Surg.

[73] Marchegiani G, Bassi C (2021). Prevention, prediction, and mitigation of postoperative pancreatic fistula. Br J Surg.

[74] Alsfasser G, Leicht H, Gunster C, Rau BM, Schillinger G, Klar E (2016). Volume-outcome relationship in pancreatic surgery. Br J Surg.

[75] Krautz C, Nimptsch U, Weber GF, Mansky T, Grutzmann R (2018). Effect of hospital volume on in-hospital morbidity and mortality following pancreatic surgery in Germany. Ann Surg.

[76] Ghaferi AA, Birkmeyer JD, Dimick JB (2009). Variation in hospital mortality associated with inpatient surgery. N Engl J Med.

[77] Strobel O, Lorenz P, Hinz U, Gaida M, Konig AK, Hank T (2020). Actual five-year survival after upfront resection for pancreatic ductal adenocarcinoma: who beats the odds?. Ann Surg.

[78] Neoptolemos JP, Palmer DH, Ghaneh P, Psarelli EE, Valle JW, Halloran CM (2017). Comparison of adjuvant gemcitabine and capecitabine with gemcitabine monotherapy in patients with resected pancreatic cancer (ESPAC-4): a multicentre, open-label, randomised, phase 3 trial. Lancet.

[79] Conroy T, Hammel P, Hebbar M, Ben Abdelghani M, Wei AC, Raoul JL (2018). FOLFIRINOX or gemcitabine as adjuvant therapy for pancreatic cancer. N Engl J Med.

